# Prolonged dialysis during *ex vivo* lung perfusion promotes inflammatory responses

**DOI:** 10.3389/fimmu.2024.1365964

**Published:** 2024-03-22

**Authors:** Julien De Wolf, Carla Gouin, Luc Jouneau, Matthieu Glorion, Antoine Premachandra, Florentina Pascale, Maxime Huriet, Jérôme Estephan, Jean-Jacques Leplat, Giorgia Egidy, Christophe Richard, Valérie Gelin, Céline Urien, Antoine Roux, Morgan Le Guen, Isabelle Schwartz-Cornil, Edouard Sage

**Affiliations:** ^1^ Department of Thoracic Surgery and Lung Transplantation, Foch Hospital, Suresnes, France; ^2^ Université Paris-Saclay, INRAE, UVSQ, VIM, Jouy-en-Josas, France; ^3^ Université Paris-Saclay, INRAE, AgroParisTech, GABI, Jouy-en-Josas, France; ^4^ Université Paris-Saclay, UVSQ, INRAE, BREED, MIMA2, CIMA, Jouy-en-Josas, France; ^5^ Department of Pulmonology, Foch Hospital, Suresnes, France; ^6^ Department of Anesthesiology, Foch Hospital, Suresnes, France

**Keywords:** lung, transplantation, *ex vivo*, ischemia-reperfusion, dialysis, *ex vivo* lung perfusion, pig

## Abstract

*Ex-vivo* lung perfusion (EVLP) has extended the number of transplantable lungs by reconditioning marginal organs. However, EVLP is performed at 37°C without homeostatic regulation leading to metabolic wastes’ accumulation in the perfusate and, as a corrective measure, the costly perfusate is repeatedly replaced during the standard of care procedure. As an interesting alternative, a hemodialyzer could be placed on the EVLP circuit, which was previously shown to rebalance the perfusate composition and to maintain lung function and viability without appearing to impact the global gene expression in the lung. Here, we assessed the biological effects of a hemodialyzer during EVLP by performing biochemical and refined functional genomic analyses over a 12h procedure in a pig model. We found that dialysis stabilized electrolytic and metabolic parameters of the perfusate but enhanced the gene expression and protein accumulation of several inflammatory cytokines and promoted a genomic profile predicting higher endothelial activation already at 6h and higher immune cytokine signaling at 12h. Therefore, epuration of EVLP with a dialyzer, while correcting features of the perfusate composition and maintaining the respiratory function, promotes inflammatory responses in the tissue. This finding suggests that modifying the metabolite composition of the perfusate by dialysis during EVLP can have detrimental effects on the tissue response and that this strategy should not be transferred as such to the clinic.

## Introduction

*Ex-vivo* lung perfusion (EVLP) has been adopted by many lung transplantation centers around the world for reconditioning marginal donor lungs. EVLP consists in ventilating and perfusing donor lungs at normothermia with an adapted oxygenized perfusing fluid including or not erythrocytes. With this process, donor lungs that present mild dysfunction can be assessed and may recover the respiratory and circulatory properties required for transplantation (1). EVLP therefore led to increasing the number of lung transplantations by about 20% (2), with similar or even better outcomes than the ones using conventional cold storage (1).

However, EVLP presents limitations. Indeed, after several hours of procedure, EVLP is systematically associated with edema formation, which compromises gas exchange and leads to deterioration of the lung functions (3). In addition, many reports emphasize that EVLP induces inflammatory responses, which have been correlated with poor outcome post-transplantation (4–7). The inflammatory response is thought to be initiated by the ischemia-reperfusion response that inevitably occurs upon organ procurement and reperfusion in the EVLP system (8). The lack of oxygen at procurement leads to a decrease of adenosine triphosphate production by the mitochondria that drive cell acidosis and dysfunction of ionic pumps, altering cell viability. Upon reperfusion, the reintroduction of O2 causes huge electronic effluxes across the mitochondrial membrane, production of reactive oxygen species (ROS) causing oxidative damages and further cell death, and the liberation of inflammatory cytokines together with damage-associated molecular pattern (DAMP) molecules including HMGB and HSP70 that all accumulate during EVLP (6, 9–11). These DAMPs may further amplify inflammation, possibly through the TLR4-MyD88-NFKB signaling pathway (12–15). Furthermore, the lung is maintained metabolically active during EVLP and accumulates metabolic wastes in the absence of detoxification by liver and kidney. Therefore, the pH decreases and some electrolytes accumulate, with negative effects on cell viability (16). Furthermore, metabolomic analyses of EVLP revealed the accumulation of by-products in the perfusion liquid of EVLP, such as lactate and pyruvate, amino acids, polyunsaturated lipids, eicosanoids, and RNA breakdowns (17, 18). These by-products can have by themselves cytokine-like and pro-inflammatory functions (19), thus possibly reinforcing the inflammatory response induced by ischemia reperfusion. For mitigating these imperfect metabolic conditions and reducing the accumulation of inflammatory molecules, the costly perfusate fluid is regularly replaced, which impacts on the economical aspect of the procedure. Importantly, while EVLP is approved to support lung for 6h in the clinical practice (20), longer durations are also desired to facilitate the operating room logistics and to optimally prepare the lung with advanced therapeutic interventions (cell or gene therapy, immunomodulation).

In order to find a solution to improve and prolong EVLP as well as to decrease its cost, dialyzers were added to EVLP machines and experimentally tested on human lungs (21) and pig (22, 23). Overall satisfying conclusions were published, with maintenance of the perfusate composition, and possibly improved cell viability (21–23). In a recent work, we also reported that the transcriptomic changes induced by acellular EVLP in a pig model (Toronto protocol) appeared similar during a 6h procedure when comparing different perfusate management techniques, that is, no perfusate modification, repeated replacement, and adult and pediatric dialysis (23). Altogether, while addition of a dialyzer on the EVLP circuit appears feasible, more biological knowledge on the dialysis effect is needed to better capture the possible risks and benefits before transfer to the clinic. In this study, we assessed the effect of dialysis during a 12h procedure and performed extended biostatistical and predictive functional genomics analyses in the lung graft. Compared with perfusate replacement, we found that dialysis, while maintaining the lung function and correcting parameters of the perfusate electrolytic and metabolic composition during 12h, induced a higher accumulation of inflammatory cytokines in the perfusate as well as a genomic profile predicting increased permeability of vasculature and enhanced cytokine signaling.

## Material and methods

### Ethics

The pig experiments were performed in accordance with the EU guidelines and the French regulations (DIRECTIVE 2010/63/EU, 2010; Code rural, 2018; Décret n°2013-118, 2013). The procedures were approved by the Ministry of Higher Education and Research and the experiments were approved by the COMETHEA ethic committee under the APAFIS authorization number APAFIS#14942-2018020720215374 v4. The surgery was conducted at the Medical Imaging in Animal platform (accreditation B78-322-2) and the animals were hosted at the Animal Genetics and Integrative Biology unit at INRAE-Jouy (accreditation C78-719).

### Animals and lung extraction procedures

Nine pigs (Large-White, 50 ± 5 kg) provided the lungs that were harvested using a non-heart-beating donor model with a 10-min no touch, as described in details by us before (23).

### EVLP and dialysis procedures

After subjecting the lungs to 1h of cold ischemia, a 12h EVLP procedure was conducted according to the Toronto protocol (24). The circuit was infused with 1.5 L of Steen^®^ solution supplemented with 1 g methylprednisolone, 1.5 g cefuroxime, and 15000 UI heparin. A flow rate equivalent to 40% of the theoretical cardiac output was applied at 34°C. The pig lungs were divided into two EVLP groups, one named Gold Standard (GS, five pigs) in which 500 ml Steen^®^ solution was replaced every 2h, and the other named Pediatric Dialysis (PD, four pigs) in which a Continuous Veno-Venous Hemodialysis mode applied on the circuit as detailed in (23). The overall EVLP with dialyzer circuit is presented in Additional file 1. The dialysis bath, based on Phoxilium^®^ (Baxter, Deerfiled, IL, USA), was adjusted with suitable electrolytes. An AVpaed pediatric membrane (Fresenius, Bad Homburg vor der Höhe, Germany) featuring an effective surface area of 0.2 m^2^ and a cutoff of 30 kDa was utilized at a flow rate of 50 ml/min. Substances below 30 kDa can be eliminated with this membrane, while essential plasma proteins such as albumin constituents are retained. This pediatric membrane with a low surface area and used in an exclusive diffusive mode was chosen to minimally interfere on the filtration process. In a previous study with a so-called adult dialysis process, we used an Emic 2 membrane (Fresenius) with an effective surface area of 1.8 m^2^ and a cutoff at 40 kDa, and we applied a flow rate of 800 mL/h and 80 mL/min (23). The cutoff of the pediatric membrane is better adapted to the EVLP conditions, avoiding the depletion of albumin and dextran from the Steen fluid.

The PD group included the same lungs as the ones utilized in (23) for the 6h time point. The GS group is totally distinct (see the Discussion section).

### EVLP monitoring, sample collections, and biological dosages

Throughout the EVLP process, pulmonary artery pressure, graft weight, temperature, ventilation peak pressure, ventilation plateau pressure, and compliance were systematically documented every hour. Perfusates were collected at hourly intervals and frozen at −80°C for lactate deshydrogenase (LDH) and cytokine detection. Blood gases, electrolytes, and proteins were detected on fresh perfusates. The LDH was measured with the CytoTox 96 Non-Radio Cytotoxicity Assay (Promega, Madison, WI, USA). Cytokine concentrations were assessed with a porcine cytokine magnetic bead panel kit, the Milliplex-MAP-Kit (PCYTMAG-23K-07, Millipore, Billerica, MA, USA), for the detection of TNFα, IL-8, IL-6, IL-10, IL-1a, IL-1β, and IL-1RA. The C5a complement was measured in the perfusates using Pig Complement C5a (C5a) ELISA Kit (Abbexa, Cambridge, UK). Lung biopsies were collected right after the cold ischemia period (0h), at 6h and 12h and either frozen in liquid nitrogen and kept at −70°C or stored in RNAlater (Thermo Fisher Scientific, Waltham, MA, USA) at −20°C.

### RNA extraction, quality check, and deep sequencing

Frozen lung biopsies were embedded in Tissue-Tek O.C.T. Compound (Sakura-Finetek France SAS, Villeneuve d’Ascq, France), cut in slices (five slices, 60-µm thick) from which total RNA was extracted using the Arcturus PicoPure RNA Isolation Kit (Arcturus Technologies Inc, Leesburg, USA). Alternatively, biopsies in RNAlater were placed in Trizol, homogenized with 1.4 mm ceramic beads in a Precellys 24 bead grinder homogenizer (Bertin Technologies, St Quentin en Yvelines, France), and purified using the NucleoSpin RNA kit that includes a DNAse digestion step (Macherey-Nagel, Düren, Germany). RNA was checked for quality with an Agilent 2100 Bioanalyzer using RNA 6000 Nano Kits (Agilent Technologies, Santa Clara, CA, USA). Directional RNA-seq libraries were constructed from 500 ng of total RNA using the TruSeq mRNA Stranded library prep kit (Illumina, San Diego, USA), following the manufacturer’s instructions. The quality of the libraries was assessed with an Agilent Bioanalyzer 2100, using an Agilent High Sensitivity DNA Kit. Libraries were pooled in equimolar proportions and sequenced in paired-end runs (50 nt forward-34 nt reverse) on an Illumina NextSeq500 instrument, using NextSeq 500 Mid Output kit v2. Demultiplexing has been done with bcl2fastq2 V2.2.18.12. Adapters were trimmed with Cutadapt1.15. Reads above 10 pb were kept.

### Bioinformatic analyses

The Illumina sequencing produced from 25 to 95 million reads per sample. Sequences were aligned with tophat2 (v2.0.14; options: -N 2 –read-edit-dist 2 –b2-sensitive –no-coverage-search) on the porcine transcriptome (reference Ensembl Release 101, Sscrofa11.1). Gene counts were processed using RLOG function of DESeq2 package (v1.18.1). In order to study the functional signatures of the gene expression changes between timing (12h vs. 0h and 6h vs. 0h) in the GS and PD groups (i.e. four conditions), we performed a differential expression analysis using the DESeq2 R package (v1.18.1) for paired data sets. Notably, for the GS group, the differential expression of the genes versus 0h (before EVLP initiation) was calculated differently in our previous study (23): in that study, the differential expression was calculated relatively to ten unpaired lung tissues at 0h whereas, here, the differential expression was calculated relatively to the same paired lung tissue at 0h. The differential expression analysis included an independent hypothesis weighting batch correction and a Benjamini–Hochberg correction for multiple testing. The differentially expressed genes (DEGs) were selected based on an adjusted *p*-value below 0.05 and an absolute fold change mean value superior or equal to 2 (Additional file 2). The four DEG lists were used as input into functional enrichment analysis using the Hallmark gene set collection of the MSigDB and the Reactome gene set collection. The functional enrichments were selected based on a −Log_10_ adjusted *p*-value superior to 3 and on over three contributing genes in at least one of the four conditions. As the analysis using Reactome gave redundant results with Hallmark, we selected the Hallmark results. In addition, the four DEG lists were submitted to an ingenuity pathway analysis (IPA, www.ingenuity.com, QIAGEN Silicon Valley, Redwood City, CA, edition 2019). The pre-analysis filtering on tissues and cell lines included endothelial cells, epithelial cells, fibroblasts, immune cells, and lung. The association likelihood of the DEG sets with a given pathway/function is given by *p*-values calculated with the right-tailed Fisher’s exact test, and the prediction of their activation or inhibition is given by *z*-scores (> 2 or < −2, respectively). The *z*-scores are calculated with an algorithm that integrates (i) the expression orientation of the genes in the data set that contribute to the pathway/function and (ii) the knowledge on these genes expression from experimental data published in peer-reviewed journals. The biologically relevant functions and pathways presenting at least an absolute *z*-score value > 2 and −Log_10_ *p*-value > 1.3 with over three contributing genes in one of the four conditions were selected. We also proceeded to a differential expression analysis between 12h versus 6h in the GS and PD groups (Additional file 2) and we performed an enrichment analysis using the Hallmark, Reactome, and IPA gene set collections as described above.

Finally, we performed a comparative analysis of the gene expression fold changes (FCs) at 6h (6h vs. 0h) and at 12h (12h vs. 0h) between the GS and PD groups using an unpaired and non-parametric Wilcoxson test, as the FC data did not pass a normality test. The comparison gene list was obtained from the FC comparison generating adjusted *p*-values below 0.05 with the Wilcoxon test (**Additional file 3**), and it was submitted to functional enrichment analyses using the Hallmark, the Reactome gene set collections, and IPA. The functional enrichments with −Log_10_ *p*-value > 3 and over three contributing genes were selected. As Hallmark results showed overlaps with Reactome and IPA results, only Reactome and IPA results were selected.

### Statistical analyses

The physiological, electrolytic, metabolic, C5a, and cytokine data were analyzed with the R Studio (v2023.03.1 + 446). A Shapiro test was used to evaluate the normality of the data distribution in each group and timing. The cytokine data were log_10_-transformed. When the data did not pass the normality test, a non-parametric Mann–Whitney test was used to compare the data between two groups. Alternatively, an unpaired *t*-test was used upon equal variance evaluation. The statistical methods of the bioinformatic analyses are reported in the dedicated paragraph.

## Results

### Pediatric Dialysis corrects and stabilizes electrolytic and metabolic parameters during 12h EVLP

**Figure 1:**
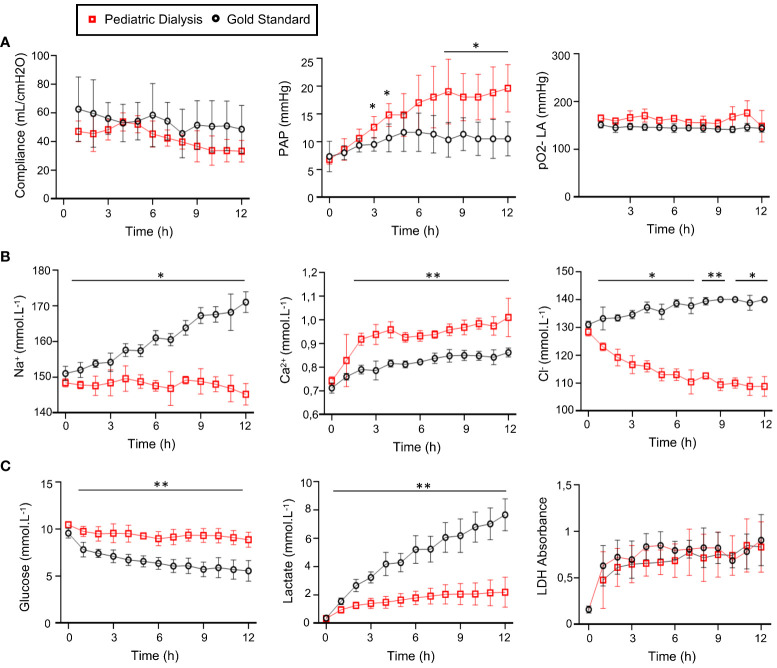
Monitoring of the physiological parameters **(A)**, electrolyte **(B)**, and metabolite **(C)** concentrations in the perfusates in the Gold Standard (five pigs) and Pediatric Dialysis groups (four pigs). **(A)** From left to right: lung compliance, pulmonary artery pressure, pO2 on FiO2 30%. **(B)** From left to right: Na^2+^, Ca^2+^, Cl^−^ concentrations. **(C)** From left to right: glucose, lactate production, lactate deshydrogenase (LDH). To compare the data between the two groups, an unpaired *t*-test was used when the data passed the Shapiro normality test. For cases that did not pass the Shapiro normality test, a non-parametric Mann–Whitney test was performed. The *p*-value classes are reported as *p < 0.05 or **p < 0.01 at specific time points. The highest *p*-value class common to several time points is reported above a line. The error bars represent standard deviations.

Based on the results of our initial study that showed no significant effect on the lung function and gene expression profiles of different perfusate managements over 6h EVLP (23), we aimed at evaluating the effect of dialysis over a longer duration, that is, 12h. In that first study, we tested two methods of dialysis, referred to as adult or pediatric, that differed by the type of dialysis membrane and the applied flow rates. After 6h procedure, the Adult Dialysis EVLP (40 kDa cutoff membrane) led to important edema accumulation in the organ, possibly due to loss of components of the Steen perfusate through the adult dialysis membrane and was therefore abandoned. Conversely, the weight gains at the end of the procedure, which can be used as proxy to estimate edema, were similar in the PD (30 kDa cutoff membrane) and in the GS procedures (222 ± 107 gr for PD and 186 ± 106 gr for GS, mean ± sd); therefore, the effects of these two procedures were compared in further analyses. The compliance and gas exchanges showed no differences between the PD and GS groups (**Figure 1A**). The pulmonary artery pressure was significantly higher in the PD group at several time points (**Figure 1A**, *p* < 0.05). The Na^2+^ and Cl^−^ ionic concentrations remained low in the PD group and increased in the GS group, and conversely the Ca^2+^ levels increased in the PD group more than in the GS group (**Figure 1B**, *p* < 0.01). The glucose and lactate levels remained stable in the PD group while the glucose steadily decreased and the lactate increased overtime in the GS group, indicating a better control of the glucose metabolism in the PD group (**Figure 1C**, *p* < 0.01 for lactate and glucose). The LDH levels did not differ between the two groups over 12h, supporting that PD did not increase cell death in the lung (**Figure 1C**). Overall, the improvement of composition of the perfusate induced by the PD process that we observed at 6h was also confirmed at 12h, with maintenance of respiratory functions.

### Pediatric Dialysis enhances the concentration of several inflammatory cytokines in the perfusate and enhances the expression of cytokine genes in the lung tissue

**Figure 2:**
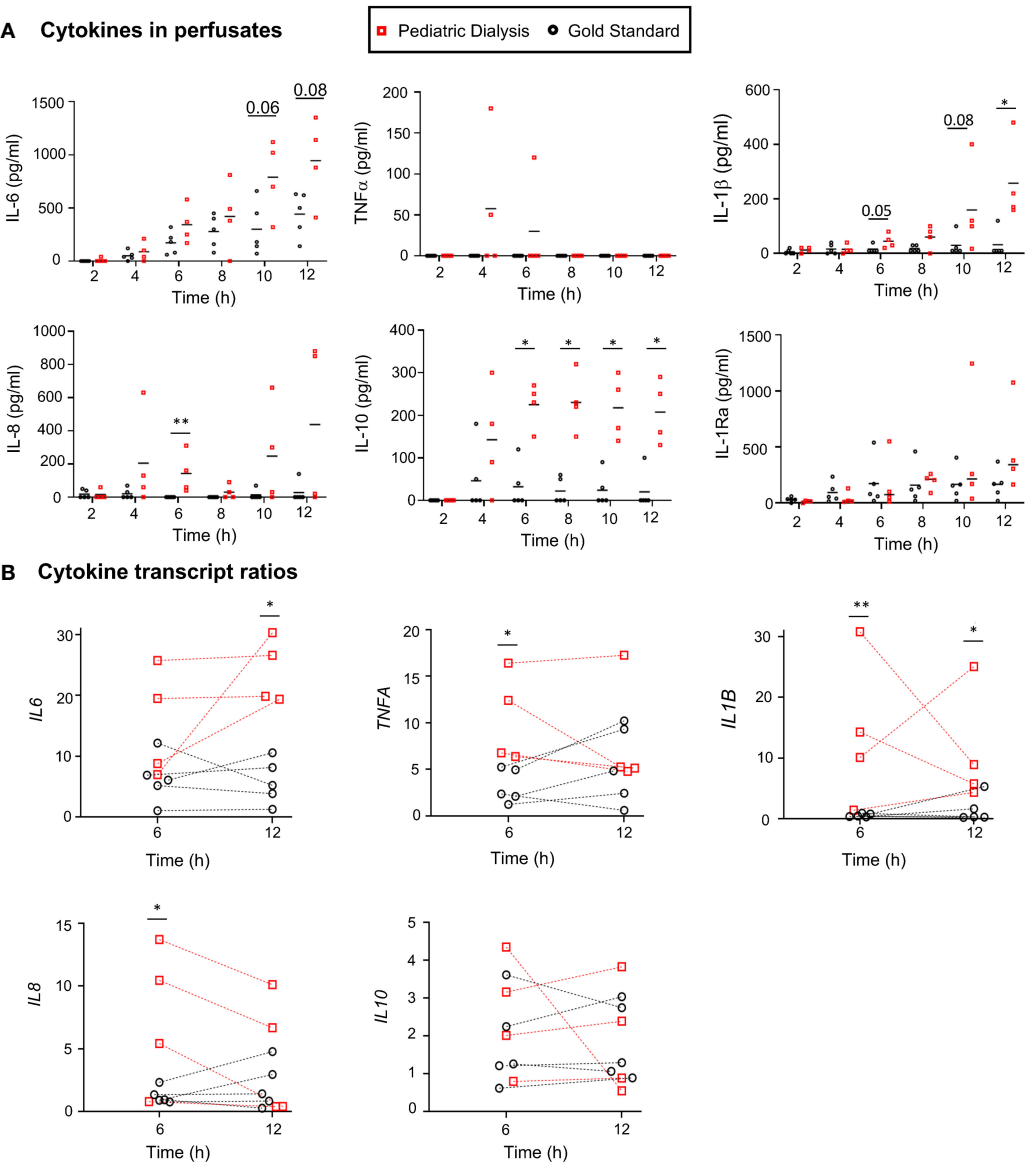
Cytokine protein and transcript analyses in the Gold Standard and Pediatric Dialysis groups. **(A)** Cytokines were detected in the perfusates using a porcine cytokine magnetic bead panel kit. At specific time points, when the data followed a normal distribution after Log_10_ transformation, a *t*-test was used to compare the data between the two groups; alternatively, a non-parametric Mann–Whitney test was used, *p < 0.05, **p < 0.01 or exact value in case of tendency. **(B)** The cytokine gene expression ratios were established from the RNA-seq gene counts at 6h and 12h divided by the gene counts at 0h. The same statistical analysis was done as in A at specific time points.

We measured inflammatory cytokine levels in the perfusate, and we observed that cytokine levels tended to be higher in the PD group (**Figure 2A**); this is notably the case for interleukin-6 (IL-6) at 10h (*p* = 0.06) and 12h (*p* = 0.08), IL-8 at 6h (*p* < 0.01), IL-1β at 6h (*p* = 0.05), at 10h (*p* = 0.08) and at 12h (*p* < 0.05), and IL-10 at all timing from 6h (*p* < 0.01). Higher levels of IL-6 and IL-10 at 6h were also found in the PD group in our first study (23). No cytokine could be detected in the dialysis bath. In order to determine whether this finding could be related to increased gene expression in the tissue, we proceeded to RNA-seq analysis of the lung tissue at 0h, 6h, and 12h and compared the fold changes (FC, 6h vs. 0h and 12h vs. 0h) of the different cytokine mRNA counts between the PD and GS groups (**Figure 2B**). We observed higher levels of upregulated transcripts in the PD group for *IL-6* at 12h, *IL-1B* at 6h and 12h, and tumor necrosis factor*–*α (*TNFA*) at 6h. No significant difference of transcript levels was found between the two groups in the case of the *IL-10* gene.

Altogether, the EVLP with PD increased the expression of several cytokine genes in the lung tissue and led to higher levels of several cytokine proteins in the perfusate, despite the process of dialysis through a 30 kDa membrane.

### Pediatric Dialysis and Gold Standard both activate gene expression pathways related to inflammatory responses and activation of antigen-presenting cells

**Figure 3:**
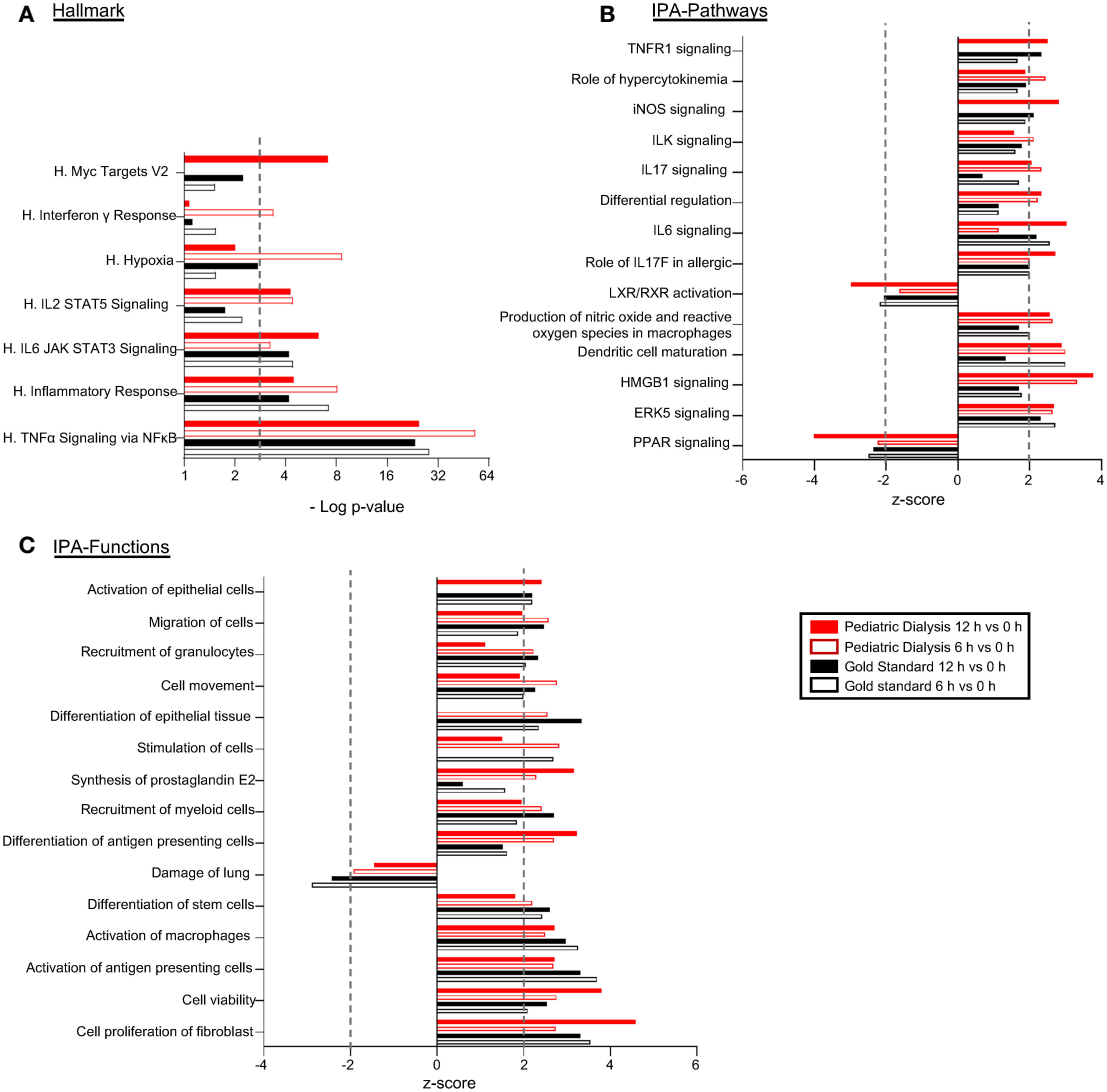
Functional enrichments and predictions of modulated pathways/functions induced by EVLP in the Gold Standard and Pediatric Dialysis groups. Differentially expressed gene lists (DEGs) were established from the RNA-seq results of lung tissue undergoing EVLP at 6h versus 0h and at 12h versus 0h, in the Gold Standard and Pediatric Dialysis groups. **(A)** The DEGs were subjected to a functional enrichment analysis using the Hallmark gene sets. The functional enrichments were selected based on a −Log_10_ adjusted *p*-value superior to 3 and over three contributing genes in at least one of the four conditions. **(B, C)** The DEGs were loaded and processed through the IPA core analysis for identification of predicted activated of inhibited canonical pathways **(B)** and functions **(C)** that were selected based on their biological relevance, absolute value of *z*-score superior to 2 with a −Log_10_ *p*-value superior to 1.3 and over three contributing genes, in at least one of the four cases. The 15 top functions/pathways are shown. The contributing genes to the functional enrichments are reported in **Additional file 4**.

In order to examine the overall modulation of gene expression induced by EVLP in the GS and PD conditions, the DEG lists between 6h versus 0h and 12h versus 0h generated from the global RNA-seq results were submitted to a functional enrichment analysis (see the Material and methods section, **Additional files 2, 4**, **Figure 3A**). The enrichment analysis using the Hallmark data base showed that the TNF-α signaling via NFkB and the Inflammatory response were the top-enriched pathways (adjusted *p*-value < 0.001) in both groups at 6h and 12h. The enrichment of these inflammation-related functions was associated to the significant upregulation of the *CSF3, IL-6, TNFA*, *TNFAIP3, MAP2K3, MAFF, REL* genes in all groups and timings (Additional files 2 and 4, **Figure 3A**). The DEG lists and their statistical parameters were uploaded to the Ingenuity Systems (IPA), software that predicts modulation of functions and pathways by integrating the orientation of gene expression modulation and a scientific literature-based database. The downstream analysis of IPA predicted the activation of the ERK5-, HMGB-, IL-6-, and IL-17 signaling pathways, the inhibition of the PPAR pathway, as well as the activation of production of nitric oxide and ROS in macrophages- and of dendritic-cell maturation pathways (absolute *z*-score > 2), both in the GS and PD groups (Additional file 4, **Figure 3B**). IPA also showed the activation of cell proliferation and viability, activation of antigen-presenting cells and of macrophages, reduction of lung cell damage, and activation of cell movement (absolute *z*-score > 2 (**Figure 3C**). Finally, we submitted the DEG lists between 12h and 6h (Additional file 2) to functional enrichments. The sole significant functional enrichment was found in the case of the PD group using the Reactome data base, which revealed enrichment for the metabolism of RNA (Additional file 5).

Overall, the functional genomic analysis of the PD and GS group RNA-seq data predicted stimulated viability and activation of innate immune responses in both groups at 6h and 12h EVLP.

### Pediatric Dialysis stimulates higher expression of gene modules related to endothelial activation at 6h and cytokine signaling and metabolism of RNA at 12h

**Figure 4:**
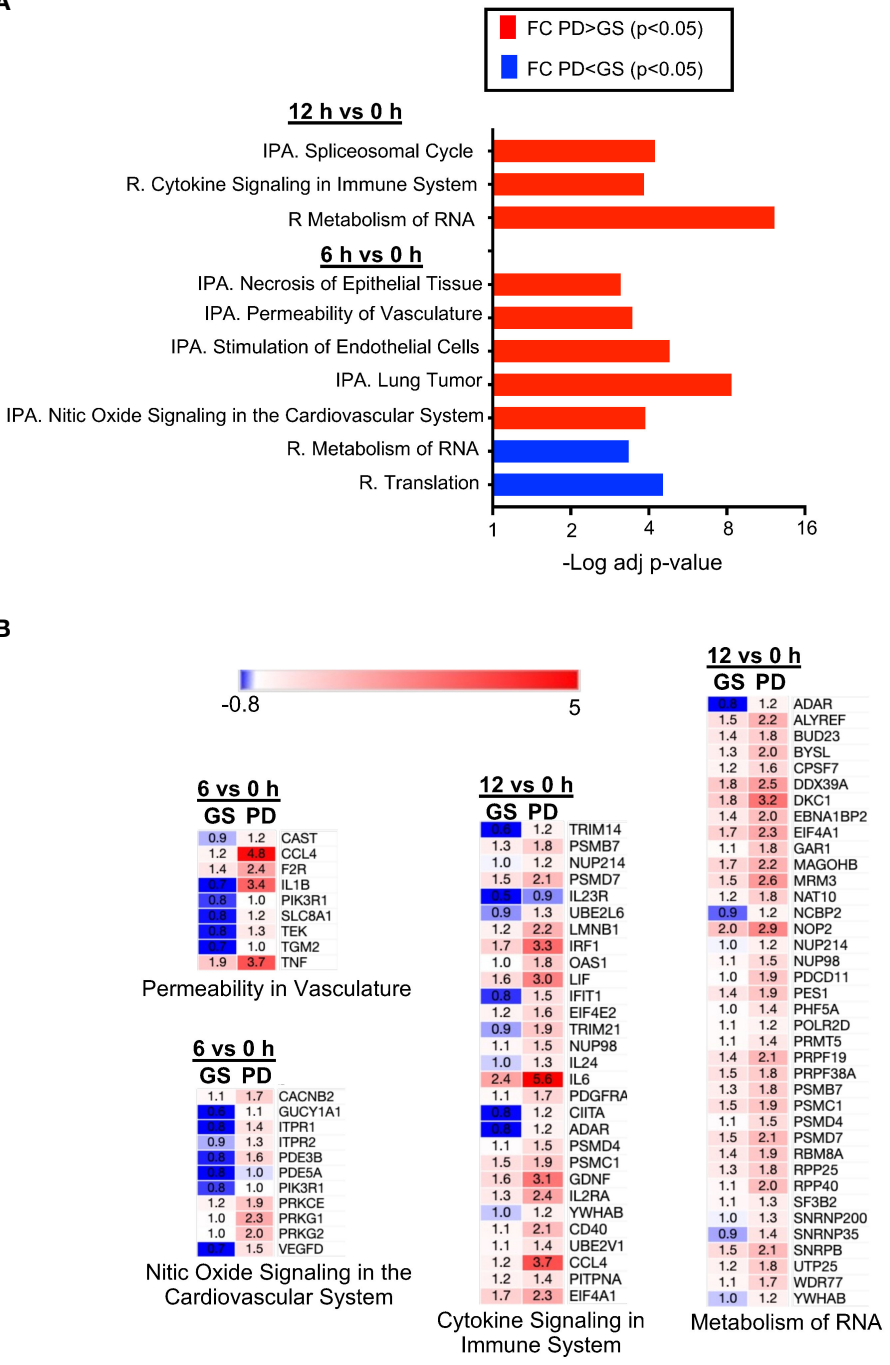
Functional enrichments of the gene expression comparisons between the Gold Standard and Pediatric Dialysis groups. **(A)** A Wilcoxson test was performed to compare the expression fold change (FC) at 6h and 12h between the two groups. An adjusted *p*-value of 0.05 was used to filter the genes of the expression ratio comparisons and the resulting gene lists were subjected to an enrichment analysis using the IPA and Reactome datasets. The enriched functions with −Log10 *p*-values > 3 and > 3 contributing genes were selected. The enriched functions in the comparison of FC that is higher in the Pediatric Dialysis group are in red; they are blue when the comparison of FC is higher in the Gold Standard group. **(B)** The mean gene fold changes of the contributing genes of some of the enriched functions shown in A are illustrated as an heat map, in blue when down-modulated and in red when upregulated at the reported timing versus 0h.

In several instances, the *z*-scores of the IPA downstream analysis of the DEGs between 6h versus 0h and 12h versus 0h appeared higher in the PD than in the GS group (**Figure 3C**) and **Figure 1** indicated higher expression ratios of several cytokine genes in the PD group. We thus aimed at comparing the gene expression FC at 6h and at 12h between the GS et PD groups, using a non-parametric Wilcoxon statistical test. We investigated the functional enrichment of the genes with statistically different FC between the two groups, using IPA and Reactome databases (**Additional file 3**, **Figure 4A**). At 6h, the genes with higher FC in the PD group were enriched for nitric oxide signaling, stimulation of endothelial cells and permeability of vasculature, with contributing genes including *C-C Motif Chemokine Ligand 4 (CCL4)*, *IL-1B*, *TNFA*, and *Endothelial Tyrosine Kinase (TEK)* (**Figures 4A, B**). At 12h, the genes with higher FC in the PD group had enriched profiles related to cytokine signaling in immune system, with higher expression FC of *Interferon Regulatory Factor 1 (IRF1), LIF Interleukin 6 Family Cytokine (LIF), IL-6, IL24, Class II Major Histocompatibility Complex Transactivator (CIITA), CD40, CCL4* (**Figures 4A, B**). Finally, an enrichment for metabolism of RNA was found for the FC that were higher in the PD than in the GS group at 12h (**Figures 4A, B**).

Altogether, the comparison of the FC between the GS and PD group shows that endothelial cell activation at 6h and cytokine signaling gene pathways at 12h were statistically more engaged in the PD group than in the GS group.

### The dialysis process does not lead to higher C5 complement activation in the circuit

**Figure 5:**
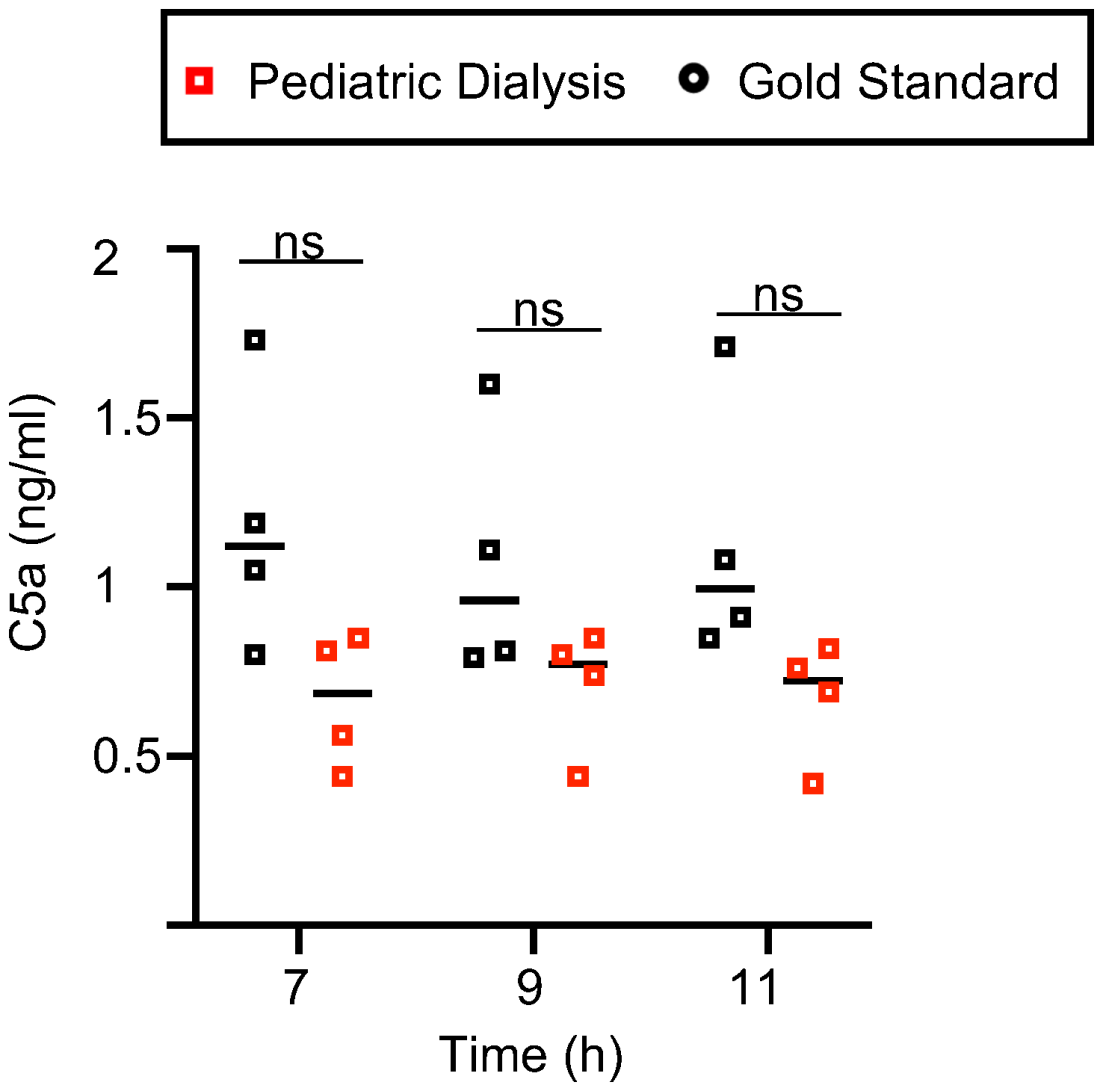
C5a levels in the Gold Standard and Pediatric Dialysis groups. The porcine C5a was detected by ELISA in the perfusates. The raw data followed a normal distribution with equal variance, and were compared between the two groups with an unpaired *t*-test. No statistically significant difference was found.

Dialysis membrane can lead to complement activation, notably of C5 (25). Complement is produced mainly by the liver, but it can also be produced by lung cells, in particular by immune cells. Activated complement C5a, if released in the circuit and activated upon membranes, might recirculate to the lung and activate the inflammatory response in the tissue, leading to a feed forward loop. We tested this hypothesis by measuring C5a in the perfusates at 7h, 9h, and 11h. While C5a was indeed detected in the two groups (range: 0.8–1.7 ng/ml in the GS group and 0.4–0.85 ng/ml in the PD group), there was no statistically significant difference of release in the two groups, and we even observed a tendency for lower levels upon dialysis (**Figure 5**). Therefore, C5 complement activation occurs during EVLP, but it does not explain the higher inflammatory response induced by the dialysis process.

## Discussion

We report here that a dialysis circuit added to the Toronto protocol better balanced the electrolyte and metabolite composition of the perfusate than the standard of care periodic replacement of the perfusate during a 12h procedure but led to increased accumulation of inflammatory cytokines in the perfusate and higher gene activation related to endothelial activation at 6h and cytokine signaling pathways at 12h.

The transcriptomic analysis revealed the prediction of activation of the ERK5-, HMGB-, IL-6-, and IL-17 signaling pathways and inflammatory responses in both GS and PD groups, similarly to what we previously published for the 6h time point in our previous study (23). As stated in the Material and methods section, the PD group is the same in the two studies, except that the sole 6h time point was analyzed in our first study (23), whereas the GS group is totally distinct between the two studies. Furthermore, the differential expression of the genes versus 0h (before EVLP initiation) was calculated differently in the two studies: in the first study (23), the differential expression was calculated relatively to ten unpaired lung tissues at 0h whereas, here, the differential expression was calculated relatively to the same paired lung tissue at 0h (see Methods). Most conclusions of the DEG functional enrichments are similar between the two studies, supporting the robustness of the results. However, the results of the present study are stronger as they include paired analyses, and they were refined by a comparative gene expression fold-change analysis (see below). Additional differences between the groups may have been obtained with a higher number of subjects.

While metabolites and electrolytes steadily increased in the GS group over 12h, we could not detect significant modification of the transcriptome between 6h and 12h. These results confirm the finding obtained with human lungs undergoing EVLP for 12h, using microarray technology (5). However, in the PD group, genes related to RNA metabolism were differentially expressed at 12h versus 6h, suggesting a higher global cellular activity over time in this group. However, bulk analysis of RNA-seq may mask effects of prolonged EVLP on specific lung subsets; therefore, single-cell RNA-seq technology could be used to refine these conclusions and better evaluate the suspected alteration that EVLP generates overtime, given the absence of systemic regulation (renal, hepatic, pancreatic, and neurohormonal). Furthermore, additional biological metrics could be included to complement our genomic study of the dialysis effects on the lung response, such as metabolomic parameters (17), cell death evaluation (26), measurement of stress molecule release [HMGB1 (10), HSP70 (11)], evaluation of mitochondrial damages [release of mitochondrial DNA (27)], detection of NETosis (28), of endothelial molecule shedding [VCAM, glycocalyx fragments (29)] and of enzymatic activities [myeloperoxidase (30)]. Indeed, a comprehensive biometric profile would be valuable to evaluate the global biological condition of lungs post-EVLP.

The important and new finding of the present study resides in the significantly higher cytokine signaling pathway observed in the PD than in the GS group, particularly at 12h. This finding was revealed by the comparative gene expression fold-change analysis that could not be done in our previous study (because the 0h samples were not available in the GS group) and also by extending the EVLP to a 12h duration. Of note, in (23), higher levels of IL-6 and IL-10 were obtained in the perfusate, a finding that is also confirmed here and extended to IL-8 and IL-1β. However, the higher levels of cytokines in the perfusate of the PD group exhibited variable degree of statistical significance, depending on timing. A higher sensitivity could have been reached by concentrating the perfusates or by measuring the total cytokine content in biopsies. Nevertheless, higher levels of inflammatory cytokines (IL-6 and IL-10) in the perfusates of EVLP purified by dialysis were also reported by another group, with human lungs declined for transplantation, although this finding was not emphasized (21).

The prediction for higher activation of endothelial cells and nitric oxide signaling in the PD group detected at 6h may be consistent with the higher pulmonary arteria pressure that was observed in the PD group in this study as well as in the previous one (23). Indeed, endothelial gene activation induced by PD might lead to increased vascular resistivity, leading to higher pulmonary arteria pressure. However, this finding was not obtained in the human study with dialysis on the EVLP circuit (21).

Several hypotheses can be proposed to explain the higher inflammatory responses induced by PD versus GS. We excluded the hypothesis that C5 activation by the dialysis membrane could be involved. PD also leads to a modified ionic/metabolite balance that may affect inflammatory response regulation. For instance, lactate that acts through the G protein–coupled receptor 81 (GPR81) to regulate immune cells (31) may participate to the control of the inflammatory response during EVLP as lactate is filtered out by dialysis. While it appears that C5a is probably partially filtered out because its levels tended to be lower in the PD group, other compounds such as vitamin C, inhibitors of complement, and soluble receptors of cytokines that mitigate inflammation may also be filtered out. The levels of cytokines in the perfusate would have been expected to decrease by being filtered out through the 30 kDa pore of the membranes, as the molecular weight of the tested cytokines lie between 8 and 20 kDa in their monomeric form. However, in patients, hemodialysis and filtration are known to be poorly effective at decreasing inflammatory cytokines from the circulation (32). In particular, IL-10, TNFα, and IL-6 are not effectively cleared with these methods, possibly due to homo-/heteromultimerization phenomenon that leads to molecular forms of higher molecular weight than the pore size (32). In addition, in our system, heparin, which is administered in the perfusate at relatively high dose (10 UI/ml), may capture some of the cytokines and impede their elimination. Accordingly, we could not detect any cytokine in the cytokine bath. In addition, the repeated replacement of the perfusate in the GS group may also lead to reduction of cytokine concentration comparatively to the conditions of the close circuit in the PD group, especially if the cytokine is not well filtered out. In any event, the increase in inflammatory cytokine expression in the PD group may be deleterious to the outcome of transplantation. Indeed, IL-1β, IL-6, and IL-8 are biomarkers of poor outcome post-EVLP; IL-1β after 1h EVLP was predictive of the one-year survival post-lung transplantation (33), IL-8 during EVLP was associated with grade 3 primary graft dysfunction (7), the combination of IL-6 and IL-8 in an inflammatory score predicted the transplantation outcomes (34) and, recently, a machine learning approach including IL-6 and IL-8 measurements in the perfusion liquid could predict the post-transplant outcomes with high confidence (AUROC: 80) (35). Furthermore, removal of cytokines by adsorption reduced primary graft dysfunction in the pig model (26). Indeed, inflammatory cytokine signaling, in combination with the cell death and stress molecules such as HMGB1 liberated upon the ischemia-reperfusion response, is expected to promote activation of innate cells including neutrophils and monocyte/macrophages which are key cell types driving primary graft dysfunction and rejection events (36–38).

Our study includes several limitations and potential avenues can be suggested to facilitate clinical applications. First, it remains possible that different results could have been obtained with other dialysis protocols (type of membrane, dialysis bath, and flow rate). The key attributes of dialysis membranes are their molecular permeability (pore size/cutoff, sieving coefficients for different molecules), adsorptive and biocompatibility properties. Here, we used a poly-sulfone membrane (AVpaed) with a 30 kDa cutoff, with a low surface area, in an exclusive diffusive mode, to minimally interfere on the filtration process. Alternative membranes with better adsorption properties, for instance polymethylmethacrylate or AN69-types (copolymer of acrylonitrile-co-methallyl sulfonate) might have exhibit better clearance of pro-inflammatory cytokines, along with other molecules such as endotoxin or HMGB1 (39, 40). Expanding on this idea, Cytosorb^®^ adsorbers placed on the EVLP circuit showed their benefits in reducing inflammatory cytokines during EVLP, reducing cell death and improving short-term transplantation outcomes, and they also corrected for the ionic and metabolic imbalances (26, 41). Second, the components depleted by the dialysis process and/or by the metabolizing lung could be supplemented in the perfusion fluid. For instance, vitamin C (42) and L-analyl-L-glutamine (43) supplementation as well as total parenteral nutrition showed their benefits on regular EVLP results (44). Noteworthy is the Toronto team short communication reporting that dialysis and the combination of dialysis with total parenteral nutrition permitted to prolong EVLP to 36h with excellent outcome upon short term transplantation (45). Finally, the lungs of the GS and PD should be grafted in order to confirm that the predicted PD-engendered increased cytokine gene pathway activation leads to deleterious outcomes as compared to the standard protocol with the regular replacement of the costly Steen^®^ perfusate.

While the pig model is considered as the most suitable biomedical model for assessing and optimizing EVLP (46), pig and human lungs may present different types of responses, due to possible species-specific responses. Interestingly, in a previous work, we reported that 11 of the 15 top modulated genes during human EVLP were found similarly modulated during pig EVLP (23), supporting the translational value of the pig model for EVLP. Nevertheless, new dialysis protocols, assessed in the pig model, should be confirmed with human lungs.

## Conclusion

We document that perfusate clearance with addition of dialysis on the EVLP circuit stabilizes electrolytic and glucose metabolic parameters over a 12h duration with maintenance of respiratory functions but promotes the expression of inflammatory cytokine gene modules in the lung parenchyma that might have negative effects on the lung transplantation outcomes. We propose that dialysis may remove protective metabolites that are synthetized during EVLP and/or favors the accumulation of cytokines generating a pro-inflammatory feed-forward loop. This work emphasizes the interest of functional genomics to analyze the biological response to EVLP and its modifications. Although presenting seducing results in initial works, dialysis in EVLP should be further evaluated and improved before translation to the clinic. Membranes with higher adsorption properties and addition of nutrients could be considered to improve the system.

## Data availability statement

The datasets presented in this study can be found in online repositories. The names of the repository/repositories and accession number(s) can be found below: GSE241921 (GEO).

## Ethics statement

The animal study was approved by comité local d’éthique en expérimentation animale COMETHEA, France. The study was conducted in accordance with the local legislation and institutional requirements.

## Author contributions

JD: Conceptualization, Investigation, Methodology, Writing – review & editing. CG: Investigation, Methodology, Writing – review & editing, Formal analysis, Visualization. LJ: Formal analysis, Investigation, Methodology, Writing – review & editing, Conceptualization. MG: Conceptualization, Investigation, Methodology, Writing – review & editing. AP: Conceptualization, Investigation, Methodology, Writing – review & editing. FP: Investigation, Methodology, Writing – review & editing, Formal analysis, Visualization. MH: Formal analysis, Investigation, Methodology, Writing – review & editing. JE: Formal analysis, Investigation, Methodology, Writing – review & editing. J-JL: Investigation, Writing – review & editing. GE: Investigation, Writing – review & editing. CR: Investigation, Writing – review & editing, Methodology. VG: Investigation, Methodology, Writing – review & editing. CU: Investigation, Methodology, Writing – review & editing, Formal analysis. AR: Writing – review & editing, Conceptualization. ML: Conceptualization, Writing – review & editing, Formal Analysis, Investigation, Methodology. IS-C: Conceptualization, Formal analysis, Investigation, Methodology, Supervision, Validation, Writing – original draft. ES: Conceptualization, Funding acquisition, Investigation, Methodology, Project administration, Resources, Supervision, Validation, Writing – review & editing.
