# Supplementary material for: Prolonged dialysis during ex vivo lung perfusion promotes inflammatory responses
Source: Front Immunol. 2024 Mar 22;15:1365964. doi: 10.3389/fimmu.2024.1365964 (PMC10995259; doi:10.3389/fimmu.2024.1365964)
Supplement: Supplementary file 1 [file DataSheet_1.zip › Additional file 1.PPTX]

## Slide 1
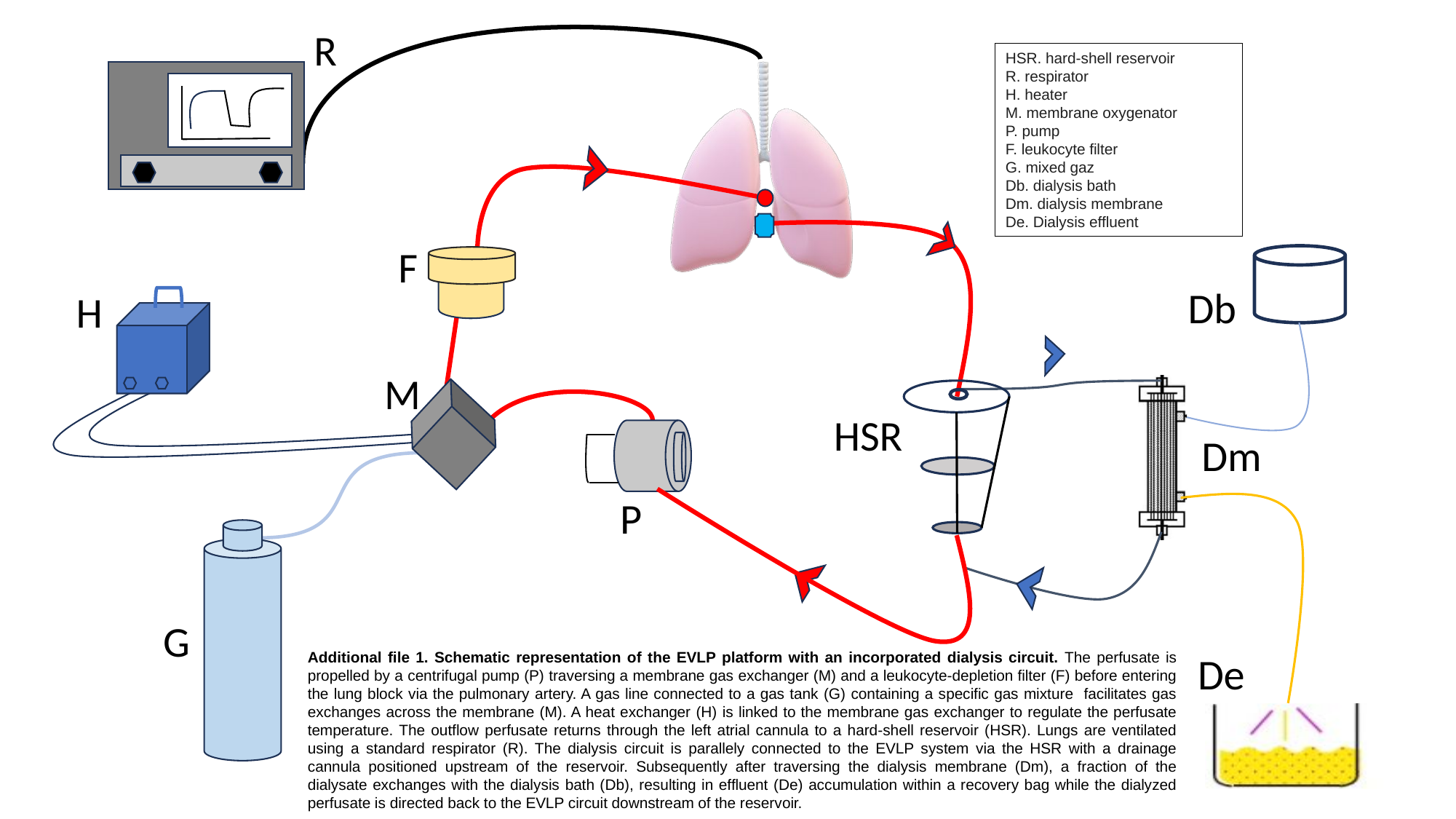

R
HSR. hard-shell reservoir
R. respirator
H. heater
M. membrane oxygenator
P. pump
F. leukocyte filter
G. mixed gaz
Db. dialysis bath
Dm. dialysis membrane
De. Dialysis effluent
F
Db
H
M
HSR
Dm
P
G
De
Additional file 1. Schematic representation of the EVLP platform with an incorporated dialysis circuit. The perfusate is propelled by a centrifugal pump (P) traversing a membrane gas exchanger (M) and a leukocyte-depletion filter (F) before entering the lung block via the pulmonary artery. A gas line connected to a gas tank (G) containing a specific gas mixture facilitates gas exchanges across the membrane (M). A heat exchanger (H) is linked to the membrane gas exchanger to regulate the perfusate temperature. The outflow perfusate returns through the left atrial cannula to a hard-shell reservoir (HSR). Lungs are ventilated using a standard respirator (R). The dialysis circuit is parallely connected to the EVLP system via the HSR with a drainage cannula positioned upstream of the reservoir. Subsequently after traversing the dialysis membrane (Dm), a fraction of the dialysate exchanges with the dialysis bath (Db), resulting in effluent (De) accumulation within a recovery bag while the dialyzed perfusate is directed back to the EVLP circuit downstream of the reservoir.
